# Understanding the Role of Estrogen Receptor Status in PRODH/POX-Dependent Apoptosis/Survival in Breast Cancer Cells

**DOI:** 10.3390/biology10121314

**Published:** 2021-12-10

**Authors:** Sylwia Lewoniewska, Ilona Oscilowska, Antonella Forlino, Jerzy Palka

**Affiliations:** 1 Department of Medicinal Chemistry, Medical University of Bialystok, Kilinskiego 1, 15-089 Bialystok, Poland; sylwialewoniewska123@wp.pl; 2 Department of Analysis and Bioanalysis of Medicines, Medical University of Bialystok, Kilinskiego 1, 15-089 Bialystok, Poland; ilona.zareba@gmail.com; 3 Department of Molecular Medicine, University of Pavia, Viale Taramelli 3/B, 27100 Pavia, Italy; aforlino@unipv.it

**Keywords:** estrogens, advanced cancer, estrogen receptor, breast cancer

## Abstract

**Simple Summary:**

The estrogen receptor (ER) status and the availability of agonists or antagonists of these receptors determine the processes of growth, differentiation, and proliferation of breast cancer cells. Estrogens and anti-estrogenic compounds have been shown to influence breast cancer cell survival/apoptosis via action through the mitochondrial enzyme proline dehydrogenase/proline oxidase (PRODH/POX). In this review, we highlight the molecular effects of ER stimulation/inhibition in signaling pathways.

**Abstract:**

It has been suggested that activation of estrogen receptor α (ER α) stimulates cell proliferation. In contrast, estrogen receptor β (ER β) has anti-proliferative and pro-apoptotic activity. Although the role of estrogens in estrogen receptor-positive breast cancer progression has been well established, the mechanism of their effect on apoptosis is not fully understood. It has been considered that ER status of breast cancer cells and estrogen availability might determine proline dehydrogenase/proline oxidase (PRODH/POX)-dependent apoptosis. PRODH/POX is a mitochondrial enzyme that converts proline into pyrroline-5-carboxylate (P5C). During this process, ATP (adenosine triphosphate) or ROS (reactive oxygen species) are produced, facilitating cell survival or death, respectively. However, the critical factor in driving PRODH/POX-dependent functions is proline availability. The amount of this amino acid is regulated at the level of prolidase (proline releasing enzyme), collagen biosynthesis (proline utilizing process), and glutamine, glutamate, α-ketoglutarate, and ornithine metabolism. Estrogens were found to upregulate prolidase activity and collagen biosynthesis. It seems that in estrogen receptor-positive breast cancer cells, prolidase supports proline for collagen biosynthesis, limiting its availability for PRODH/POX-dependent apoptosis. Moreover, lack of free proline (known to upregulate the transcriptional activity of hypoxia-inducible factor 1, HIF-1) contributes to downregulation of HIF-1-dependent pro-survival activity. The complex regulatory mechanism also involves PRODH/POX expression and activity. It is induced transcriptionally by p53 and post-transcriptionally by AMPK (AMP-activated protein kinase), which is regulated by ERs. The review also discusses the role of interconversion of proline/glutamate/ornithine in supporting proline to PRODH/POX-dependent functions. The data suggest that PRODH/POX-induced apoptosis is dependent on ER status in breast cancer cells.

## 1. Introduction

According to the WHO (World Health Organization) epidemiological data, for 2020, 19.3 million people developed cancer, and about 10 million people died. Breast cancer was the most common malignant neoplasm in women and accounted for 11.7% of all cancers globally. WHO cites obesity as one of the main reasons for the high incidence of the disease. The recent increase in the mortality of breast cancers was due to the COVID-19 pandemic that affected both therapy and prevention of the disease [1,2]. Although several therapeutic approaches for breast cancer treatment have been established, the role of estrogen receptor (ER) status in the complex regulatory mechanisms driving apoptosis/survival of cancer cells is not fully understood. Most of the studies presented in this review were done on breast cancer cell models. Although cell line models have some limitations (e.g., inability to observe systemic phenomena), they are a powerful tool which offers several advantages. Certainly, the cell models allow to strictly control conditions of the experiment in order to establish the critical factor affecting the studied processes. They are especially helpful in case of limited availability of clinical samples or in vivo models (e.g., estradiol deficiency or estrogen receptor status). Therefore, results on cell models allow to predict the consequences of pharmacotherapeutic manipulation in human. Different treatment regimens and combinations of therapies have been tested using cell lines which have yielded interesting and potentially promising results that currently have an application value [3,4].

The presence of the ER (ER+) in breast cancers increases positive response to anticancer treatment. Moreover, a better prognosis concerns progesterone receptors (PR+) and human epidermal growth factor (HER2+) positive cancers. The absence of ER is a significant risk factor for relapse and shorter life expectancy. Some authors emphasize that at least a two-receptor ER+PR+HER- expansion profile has a better prognosis than a single-receptor profile such as ER+PR-HER- or ER-PR+HER- [3]. This is probably due to the hormonal reorganization of cellular metabolism driving pro-survival or pro-apoptotic pathways. However, the mechanisms driving apoptosis/survival are not fully understood. In this report, we provide evidence that some of the ER functions could be attributed to proline dehydrogenase/proline oxidase (PRODH/POX).

## 2. Estrogen Receptors Structure, Location and Function

**Figure 1:**
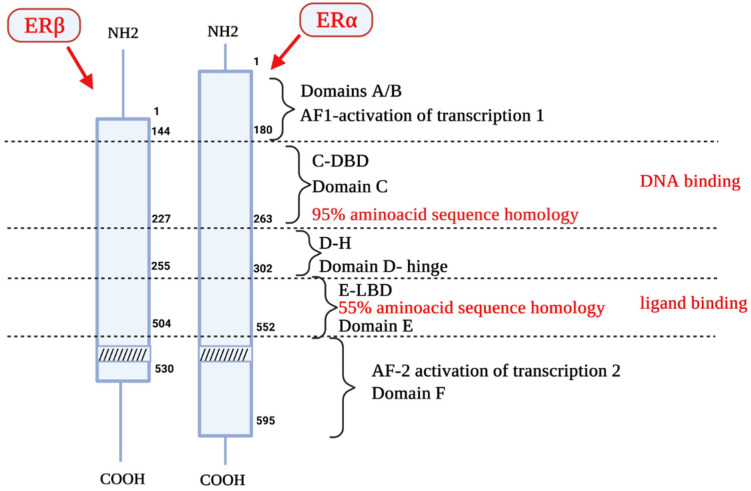
The structure of the estrogen receptor. ERα—Estrogen Receptor α; ERβ—Estrogen Receptor β; AF1—activator of transcription 1; C-DBD—DNA Binding Domain, domain C; D-H—Domain D-hinge; E-LBD—Ligand Binding Domain, domain E; AF-2—activator of transcription 2; NH2—amino-terminus, NH2—terminus, N—terminal end or amine-terminus; COOH—carboxylic terminus.

Two distinct estrogen receptor (ER) types, ERα and ERβ, are known to be encoded by two different genes located on two different chromosomes. ERα and ERβ are encoded by ESR1 (chromosome 6, region q24-q27) and ESR2 gene (chromosome 14, region q23.2). The molecular weight of ERα is 67 kDa, the ERβ isoform has 57 kDa [4]. Both types are composed of 6 functional domains named A–F [5]. Domains A and B are located at the amino terminal of the protein. The domain AF1 is able to activate gene transcription in the absence of bound ligand (e.g., the estrogen); however, the activation is weak. Domain C is responsible for receptor dimerization and binding of the ligand-receptor complex to a specific sequence on DNA. The D domain is also called the hinge. It has DNA-binding properties, and its sequence is more variable than that of the C domain. Next is the E domain, which contains a hydrophobic pocket structure called the ligand-binding domain (LBD). The E domain also enables dimerization of nuclear receptors. Some receptors also have an F domain, whose role is not fully elucidated (Figure 1) [5].

**Figure 2:**
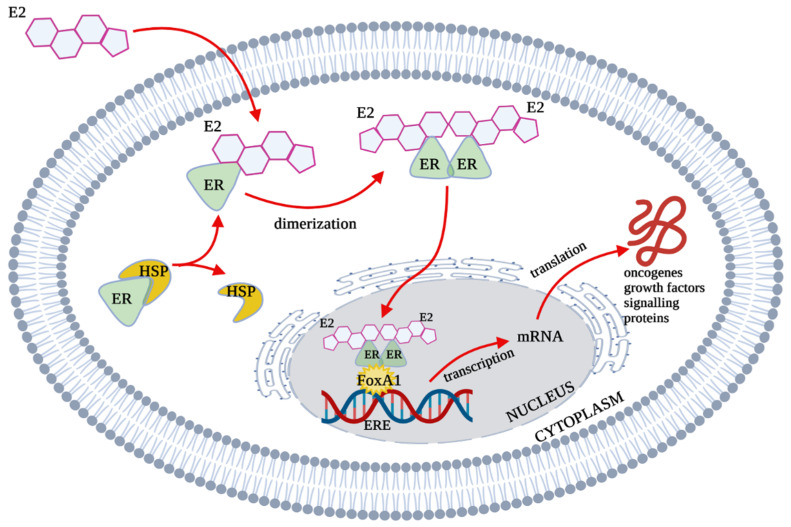
ER-dependent gene transcription. E2—estradiol; ER—Estrogen Receptor, HSP—Heat Shock Proteins; FoxA1—Forkhead box protein A1; ERE—Estrogen Response Element; mRNA—messenger RNA.

Non-active ERs occur in the cell cytosol, where they form large complexes with chaperone proteins of the HSP (Heat Shock Proteins) family. In this form, they are still inactive but capable of ligand attachment [5]. Ligand binding causes dimerization of the receptor. This process is crucial for the formation of a functional transcription factor and the regulation of gene transcription interacting with the Estrogen Response Element (ERE) (Figure 2). The molecule required for the binding of ER to DNA is FoxA1. It is a critical factor that promotes binding to chromatin [6].

The distribution of ERα and ERβ receptors in tissues and organs varies. In most tissues and organs, both types of estrogen receptors are present, while in some, only one type predominates. In the ovaries, uterus, mammary gland, kidney, adrenal gland, testes, epididymis, pituitary gland, and hypothalamus, ERα expression is higher [7,8,9] than in the urinary bladder, prostate gland, heart, and liver [10]. The highest level of ERβ expression was found in the ovary and prostate gland [11]. An important function of estrogen receptors is transcriptional and post-transcriptional regulation of cellular metabolism [12]. It has been suggested that ERα is involved in the regulation of cell proliferation, while ERβ evokes anti-proliferative and pro-apoptotic activity [13,14]. However, ERs comprise also several membranes bound receptors as G protein-coupled estrogen receptor (GPER) and Gq-coupled membrane estrogen receptor (GqmER). Recent studies revealed a functional link between all types of ERs. Interestingly, several oncogenic miRNAs have been shown to modulate the expression of ERs affecting malignant behaviour of cancer cells [15]. Moreover, a ligand-independent signaling has been reported for ERα through kind of cross-talk with epidermal growth factor or insulin-like growth factor-I [16,17]. Whether they are involved in PRODH/POX-dependent regulation of apoptosis/survival requires to be explored.

## 3. Apoptosis

Apoptosis is the process of programmed cell death, important in the development and homeostasis of multicellular organisms [18]. This process enables the elimination of damaged, old or unnecessary cells. Initiation of the apoptosis pathway is one of the possible cell responses to intracellular or extracellular action of the chemical, physical or biological factors. The external factors that cause cell damage include UV radiation, ionizing radiation, thermal shock, low availability of oxygen and nutrients, drugs, or viral and bacterial infections [19]. The internal factors are activated by oncogenes, cell cycle defects, deficiency of growth factors, energy, hormonal deregulation, etc. [20,21]. Factors inducing apoptosis contribute to the development of neurodegenerative and autoimmune diseases, growth defects, and cancer. The disturbed balance between survival and apoptosis is a common feature of cancer cells [22]. It is also the cause of resistance to chemotherapy, radiotherapy, hormonal and immune therapy [23].

Apoptosis is a precisely regulated process by several classes of proteins. The most important are caspases (a family of intracellular cysteine proteases). They are divided into initiator, implementing, and inflammation caspases. Another important protein in the apoptosis process is the family of BCL-2 proteins (Bax; Bak, Bid, Bim), which have proapoptotic, antiapoptotic, and regulatory activities [24].

Several pathways lead to the induction of apoptosis. The extrinsic pathway is initiated by binding a ligand to the death surface receptors [25]. The intrinsic pathway of apoptosis can be activated by proapoptotic factors released from mitochondria. Apoptogenic molecules that are produced during intracellular stress leads to the increase in permeation of mitochondria. Both pathways stimulate apoptosis through proteolytic cleavage of pro-caspases into active enzymes [26]. The initiator caspases include caspase-8, -9, -10, whereas caspases-3, -6, and -7 are called effector caspases [27]. They can disrupt entire cells within a few minutes.

### 3.1. The Extrinsic Apoptosis Pathway

**Figure 3:**
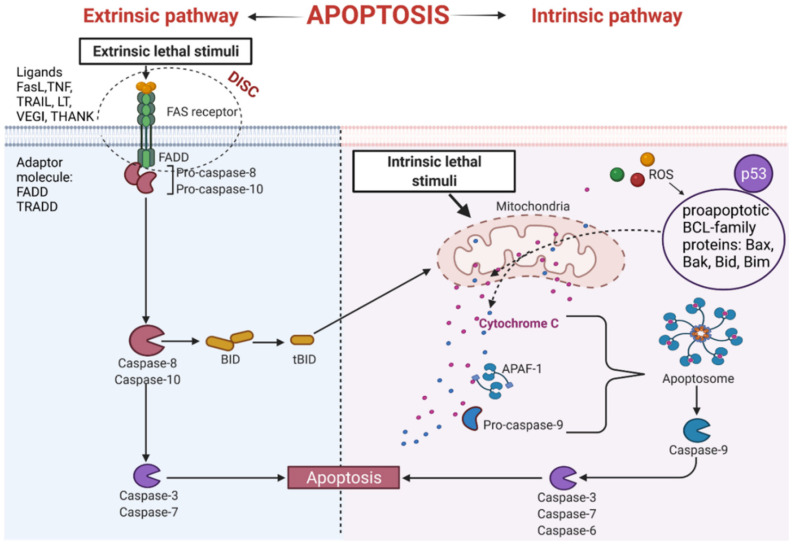
Intrinsic and extrinsic apoptotic pathway. THANK—TNF family receptor; LT—lymphotoxin; FasL—Fas Ligand; TRAIL—TNF-related apoptosis-inducing ligand; VEGI—Vascular Endothelial Growth Inhibitor; TNF—tumor necrosis factor; DISC—Death-inducing signaling complex; FADD—Fas-associated protein with death domain); TRADD—tumor necrosis factor receptor type 1-associated DEATH domain protein; p53—tumor protein p53; APAF-1—apoptotic protease activating factor-1; ROS—reactive oxygen species; Bax, Bak, Bid, Bim—proapoptotic BCL-family proteins; tBID—truncated BID.

The extrinsic process of apoptosis is induced in the cell through the signals from other cells activating the death receptor, which initiates a cascade of intracellular effector proteins [28,29]. Tumor necrosis factor (TNF) is the best-characterized protein that initiates programmed cell death [30]. The same superfamily includes ligand of TNF family receptors (THANK), lymphotoxin (LT), Fas Ligand (FasL), TNF-related apoptosis-inducing ligand (TRAIL), or the Vascular Endothelial Growth Inhibitor (VEGI) [31]. Some of them contain an intracellular death domain (DD). During protein binding to the receptors of the TNF family, the TRADD (Tumor necrosis factor receptor type 1-associated DEATH domain protein) or FADD (Fas-associated protein with death domain) adapter proteins interact with the DD region. Subsequently, the DISC complex (Death-inducing signaling complex) is formed [32,33,34]. This complex combines procaspases -8 and -10 and has autoproteolytic activation properties [35]. Cleaved caspases -8 and -10 activate the implementing caspases and initiate changes in the cell structure leading to cell death [32]. In addition, active caspases -8 and -10 activate BID (a pro-apoptotic BCL family protein), which leads to increased release of cytochrome C from mitochondria by its truncated form tBID (Figure 3).

An important apoptosis inducer is a p53 protein. This protein participates in the external and internal pathways of apoptosis. p53 interacts with BCL (B-cell lymphoma) proteins family contributing to the upregulation of mitochondrial channels and the cytochrome C efflux into the cytoplasm, activating the internal pathway of programmed cell death [36,37]. It has been established that p53 also induces genes coding for death receptors and death ligands [37].

### 3.2. The Intrinsic Apoptosis Pathway

This pathway is also called a mitochondrial pathway. It depends on energetic and metabolic processes in the cells and is induced by stress factors. These factors are oxidative stress, DNA damage, changes in cytoplasmic calcium ions concentration, and others. Furthermore, the production of reactive oxygen species (ROS) activates pro-apoptotic BCL- family proteins [38]. As a result of these reactions, the mitochondrial membrane is leaking [39], leading to the release of cytochrome C from mitochondria [38]. Released cytochrome C binds with procaspase9 and apoptotic protease activating factor-1 (APAF-1), forming apoptosome complex. The complex activates the cascade of structural changes in the cell that contribute to cell death through active forms of executive caspases such as caspase-3, caspase-6, and caspase-7 (Figure 3) [40,41].

## 4. Functional Significance of PRODH/POX in Cell Metabolism

Proline oxidase (POX), also known as proline dehydrogenase (PRODH), is a mitochondrial flavin enzyme associated with the inner mitochondrial membrane. The enzyme catalyzes proline degradation by converting this amino acid to Δ1-pyrroline-5-carboxylic acid (P5C). During this reaction, electrons are transferred via flavin adenine dinucleotide (FAD) to cytochrome C in the respiratory chain, producing ATP molecules, facilitating survival. However, when electrons are transferred directly to oxygen, that happens in specific metabolic conditions, ROS are formed, inducing apoptosis or autophagy [42,43,44,45].

Although the mechanism for switching from ATP to ROS production is not fully understood, it has been suggested that excessive rates of electron transport may contribute to ROS generation [46]. The mechanism of this process is based on mitochondrial membrane potential driving ATP synthase and ATP production and the Kadenbach mechanism (occurring at high ATP/ADP radio) that involves binding of ATP to cytochrome c oxidase (CytOx) and inhibition of the enzyme. In stress situation, ATP-dependent inhibition is switched off and CytOx activity is determined by membrane potential leading to an increase in ROS production. Another mechanism depends on the quantity of electron transfer to the Heme aa3 of CytOx and, in case CytOx is inhibited by ATP, ROS production is decreased. Whether PRODH/POX-dependent ATP/ROS generation involves the same mechanism requires to be explored. However, it has been found that PRODH/POX binds to Coenzyme Q1 (coQ1) decreasing respiratory fitness that was counteracted by N-acetyl-cysteine, suggesting that the effect was mediated by PRODH/POX-dependent ROS formation [47]. Of interest is also finding that PRODH/POX is inhibited by succinate alleviating PRODH/POX effects on respiratory fitness. It suggests that PRODH/POX-induced ATP or ROS formation is metabolic contextdependent.

Conversion of mitochondrial proline into P5C by PRODH/POX may contribute to ROS-dependent intrinsic and extrinsic apoptosis [45,48,49,50,51,52]. It has been well established that overexpression of PRODH/POX causes cytochrome C release from mitochondria to cytosol and activation intrinsic apoptotic pathway by caspases-3 and -9 [53]. However, it has been also shown that upregulation of PRODH/POX induces caspase-8 activation in the extrinsic apoptotic pathway through stimulation of TNF-related apoptosis inducing ligand (TRAIL) and death receptor 5 (DR5) [53]. Moreover, the mechanism of PRODH/POX-dependent apoptosis may involve modulation of cell signaling pathways and cell cycle regulatory processes that could induce extrinsic apoptosis. The most potent inducer of PRODH/POX activity is tumor suppressor p53. Transcriptional regulation of PRODH/POX by p53 was found in the PRODH/POX promoter, containing a p53-response element [54,55,56].

**Figure 4:**
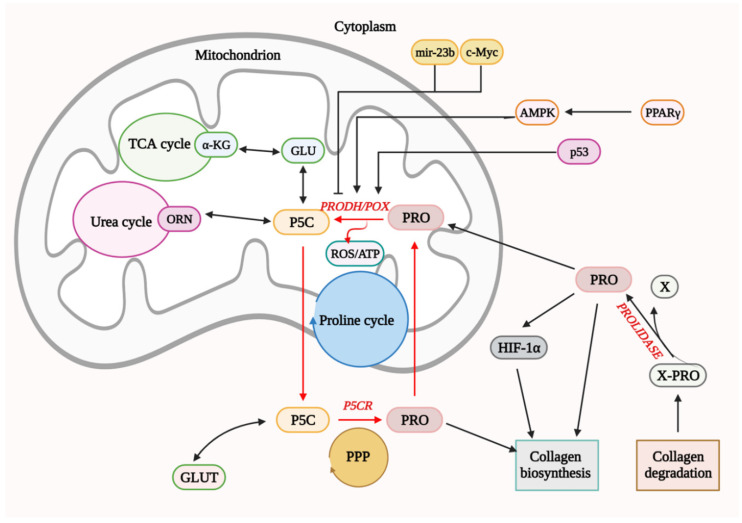
Complex regulatory mechanisms linking proline cycle, urea cycle, TCA cycle, pentose–phosphate pathway and collagen metabolism to PRODH/POX-dependent apoptosis/survival. X-Pro—amino acid-proline; PRO—proline; X—amino acid; PRODH/POX—proline dehydrogenase/proline oxidase; P5C—pyrroline-5-carboxylate; ROS—reactive oxygen species; ATP—adenosine triphosphate; GLU—glutamate; GLUT—glutamine; PPP—Pentose-Phosphate Pathway; P5CR—P5C reductase; HIF-1α—Hypoxia inducible factor 1α; TCA-cycle—The tricarboxylic acid cycle, also known as the Krebs or citric acid cycle; α-KG—α-ketoglutaric acid; ORN—ornithine; p53—TP53 or tumor protein; AMPK—AMP-activated protein kinase; PPAR γ—peroxisome proliferator activated receptor.

It seems that ATP or ROS generation depends on the metabolic context in which proline availability for PRODH/POX and proline utilization processes play a critical role. Prolidase is an important factor in providing substrate for PRODH/POX. This enzyme catalyzes the last stage of collagen degradation by releasing proline or hydroxyproline from the C-terminus of imidodi- or imidotripeptides [57,58]. Free proline could be degraded by PRODH/POX or reused for collagen biosynthesis [59]. Proline for PRODH/POX could be also derived from amino acid metabolism. The most important are glutamate and ornithine yielding P5C in reactions catalyzed by P5C synthase and ornithine aminotransferase, respectively. The generated P5C is converted into proline in reaction catalyzed by isoforms of P5CR (P5C reductases). The conversion of glutamate to the proline is catalyzed by mitochondrial PYCR ½, while the conversion of ornithine to proline is catalyzed by cytosolic PYCRL that is coupled to the Pentose Phosphate Pathway (PPP). PPP maintains a redox balance between cytosol and mitochondrion and participates in the synthesis of nucleotides [45,48]. However, the proline conversion product, P5C, can be rapidly used to synthesize glutamate by P5CDH (P5C dehydrogenase). Glutamate is, in turn, a precursor for the synthesis of α-ketoglutaric acid, which is a component of the tricarboxylic acid cycle (TCA) [48]. Proline can also be converted to ornithine, which in turn is a component of the urea cycle. These reactions link TCA and urea cycles with amino acids metabolism determining proline availability for PRODH/POX-dependent functions **(**Figure 4). However, the enzyme could be regulated by other factors. An important transcriptional regulator of PRODH/POX is the p53 protein. The presence of a response element for p53 protein in the promoter sequence of the gene coding PRODH/POX has been demonstrated. It indicates the direct participation of p53 in the transcription of PRODH/POX [49]. Among factors that inhibit the expression of PRODH/POX is the oncogenic c-MYC transcription factor that may indirectly affect PRODH/POX by stimulating expression of PRODH/POX-inhibiting factor–miR-23b [50]. This is an endogenous, non-coding small RNA fragment that has the ability to bind to the PRODH/POX 3′UTR mRNA. It has been shown that overexpression of miR-23b resulted in downregulation of PRODH/POX expression [52].

The best characterized PRODH/POX expression inducer is the peroxisome proliferator-activated receptor γ (PPAR-γ). It was shown that in the promoter sequence of the gene-encoding PRODH/POX, there are regions binding ligand-activated receptors, the so-called PPRE or PPAR-γ response element [51].

PRODH/POX participates in the induction of apoptosis by activating both extrinsic and intrinsic pathways. Activation of the extrinsic pathway requires stimulation of the transmembrane receptors containing the death domain through specific ligands [53]. PRODH/POX activates the extrinsic pathway via stimulation of geminin production. This action leads to cell cycle arrest in the G2 phase and cell apoptosis [55]. Moreover, PRODH/POX stimulates DNA damage inducible genes (GADDs).

PRODH/POX also participates in apoptosis by activating the intrinsic pathway (Figure 4). ROS formed during PRODH/POX-induced proline degradation disrupt transport in the mitochondrial membrane and affect membrane potential. In consequence, cytochrome C is released from the intermembrane space and initiates the intrinsic apoptosis pathway [54].

PRODH/POX indirectly inhibits the process of angiogenesis in tumor tissues and thus tumor growth. It degrades proline that was shown to upregulate the transcriptional activity of HIF-1α (Hypoxia-inducible factor-1) and HIF-1-dependent proteins such as vascular endothelial growth factor (VEGF) [56,60,61]. It has been documented that, in standard conditions, HIF-1α is continuously degraded by pVHL (Hippel–Lindau tumor suppressor gene product), which is a mediator in the ubiquitin pathway [56]. Proline has been shown to inhibit the degradation of HIF-1α, increasing its transcriptional activity [60]. Therefore, degradation of proline by PRODH/POX contributes to a decrease in proline level, expression of HIF-1, and angiogenesis [61].

PRODH/POX also participates in the regulation of cell proliferation. This enzyme, together with P5C reductase, forms proline cycle coupled to PPP-producing nucleotides for DNA biosynthesis (Figure 4) [62,63].

## 5. Involvement of ER Agonists in PRODH/POX-Dependent Apoptosis

ERs regulate the expression of AMP kinase (AMPK), which stimulates the activity of PRODH/POX [64,65].

The primary ligands for ER are estrogens, which represent a group of pleiotropic hormones. There are two dominant sources of estrogens in female physiology. In the pre-menopausal age, the ovaries are the principal producer of estrogens. In the post-menopausal age, when ovarian estrogen production declines, fat tissue becomes the main source. Adipocytes have a specific enzyme called aromatase, which converts testosterone to estrogen [66]. ER ligands—estrone, estriol, estradiol, and 2-hydroxy estrone—play functional roles in the physiology of the central nervous system, bones, reproductive and cardiovascular system. However, they also play an important role in carcinogenesis, stimulating cancer cell growth. These hormones act on the cancer cells by targeting the steroid receptor complex to specific DNA sequences, activating specific gene transcription. Several studies have demonstrated this mechanism using tamoxifen, a selective estrogen receptor modulator that inhibits estrogen-dependent tumor growth [67].

Estrogens regulate PRODH/POX-dependent functions at the level of ER, p53, substrate availability for PRODH/POX that is dependent on prolidase activity (proline supporting enzyme) and collagen biosynthesis (proline utilizing process), as well as HIF-1α. It seems that the most important player in determining pro-apoptotic/anti-apoptotic phenotype of cancer cells is the correlation between ERα, P53, and PRODH/POX. As pointed out in the above section, PRODH/POX is a P53-induced gene promoting apoptosis. However, ERα antagonizes P53-dependent apoptosis, promoting cell survival [68,69,70]. Based on these data, it has been established the mechanism for ERα anti-apoptotic potential, suggesting the formation of ERα-P53 complex [71]. Since ERβ was found to attenuate the complex formation, it was concluded that ERβ has pro-apoptotic activity [71]. Whether pro-apoptotic activity of ERβ undergoes through PRODH/POX that has either pro-apoptotic or pro-survival potential requires further study.

**Figure 5:**
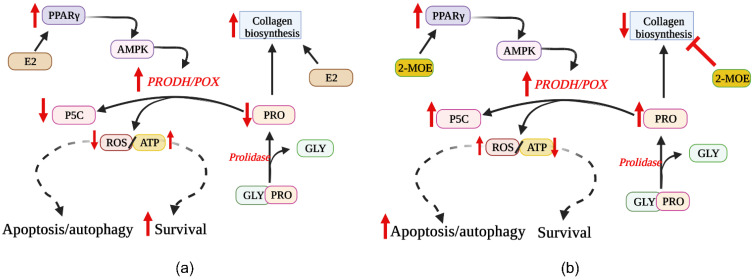
The potential mechanism of estrogen-dependent regulation of PRODH/POX-induced apoptosis. The effect of ER agonist, estradiol (**a**) and ER modulator, 2-methoxyestradiol (**b**) on PRODH/POX-dependent apoptosis in breast cancer cells. P5C—pyrroline-5-carboxylate; PRODH/POX—proline dehydrogenase/proline oxidase; ROS—reactive oxygen species; GLY—glycine; PRO—proline; PPAR γ—peroxisome proliferator activated receptor; ATP—Adenosine triphosphate; PPARγ—Peroxisome Proliferator Activated Receptor γ; AMPK—5′ adenosine monophosphate-activated protein kinase; E2—estradiol; 2-MOE—2-methoksyestradiol.

Another potential link between estrogens and PRODH/POX-dependent apoptosis is at the level of substrate availability for the enzyme. PRODH/POX is the only enzyme that degrades proline. During this process, ATP or ROS are produced (Figure 4). This amino acid could be synthesized from glutamine or ornithine. However, it is energetically unfavorable, particularly in cancer cells. Instead, proline is derived from collagen degradation products. The last step of collagen degradation is catalyzed by prolidase, releasing proline from imododipeptides [63]. The activity of prolidase may regulate proline availability for PRODH/POX-dependent functions. However, the free proline could be rapidly used for collagen resynthesis, limiting its degradation by PRODH/POX in mitochondria. Such a case may take part in MCF-7 breast cancer cells, where estradiol (independently on the ERβ/ERα status) was found to stimulate collagen biosynthesis (Figure 5a) [72,73,74]. This process limits proline availability to the proline cycle (Figure 4) and PRODH/POX-dependent functions. It has been found that ERα is involved in the upregulation of prolidase activity, suggesting that it supports proline for collagen biosynthesis [75]. Interestingly, it also induces HIF-1α transcriptional activity, contributing to the pro-survival phenotype of breast cancer cells [75].

## 6. Effects of ER Modulators on PRODH/POX-Dependent Apoptosis

Phytoestrogens are natural compounds that are ER modulators. They resemble estrogens in their structure. Phytoestrogen’s ability to binding ER induces an estrogenic response or an anti-estrogenic effect [76]. This effect depends on the concentration of the compound and the type of target tissue. Isoflavones at low concentrations have an agonist effect, and at higher concentrations, they are antagonists. Due to this feature, phytoestrogens are called selective estrogen receptor modulators (SERMs) [77]. Phytoestrogens exhibit a broad spectrum of anticancer activity. They inhibit proliferation, invasiveness and induce apoptosis of breast cancer cells. Furthermore, they modulate the activity of ROS-scavenging enzymes [78,79]. For instance, genistein is a characteristic isoflavone found in soybean and is the most abundant natural ERβ modulator. It has an affinity for both ERα and ERβ. However, it has a ninefold preferential affinity for ERβ. By regulating ERβ expression, genistein exerts anticancer effects. Numerous in vitro and in vivo studies have shown that genistein decreases cancer cell proliferation by blocking the cell cycle in the G2/M phase. Induction of apoptosis is associated with the activation of caspase-9 and downregulation of cyclin B1 [80,81].

Some studies have shown that genistein and other phytoestrogens have synergistic effects with other chemotherapeutics and enhance the efficacy of anticancer therapy. ER modulators inhibit PI3K/Akt/mTOR pathway and NF-κB activation [82]. Furthermore, Akt and NF-κB inhibition leads to downregulation of Bcl-2 protein and upregulation of Bax protein [83,84]. The possible mechanism for inhibition of the PI3K/AKT pathway and enhancement of breast cancer cell apoptosis by ER modulators (e.g., equol, biochanin A, daidzein) could be related to PRODH/POX-dependent ROS generation. It has been found that ER modulators inhibit collagen biosynthesis (proline utilization process), making proline available for PRODH/POX-dependent functions [85,86,87,88]. Whether this is the case requires to be explored. However, some line of evidence supports such a hypothesis. In contrast to 17β-estradiol (the most active estrogen in the stimulation of the collagen biosynthesis), its metabolite, 2-methoksyestradiol, has the opposite effect (Figure 5b). It inhibits collagen biosynthesis (increasing the amount of intracellular proline, the substrate for PRODH/POX) and activates PPAR-γ (stimulating PRODH/POX) [89,90]. Furthermore, 2-methoxyestradiol inhibits HIF-1α [89]. Another correlation between estrogens, collagen, and PRODH/POX was found at the level of PPAR-γ. Activation of this transcription factor is known to upregulate PRODH/POX [91]. Telmisartan, PPAR-γ ligand was found to inhibit collagen biosynthesis in breast cancer cells [92], supporting free proline for PRODH/POX-dependent functions. It is supported by several studies of other authors [93,94].

It is generally accepted that estrogens induce collagen metabolism, while anti-estrogens evoke either stimulatory or inhibitory effects, depending on the concentration of anti-estrogen, cell type and microenvironmental conditions [95,96,97]. Anti-estrogen functions are of great importance in the biology of breast cancer. Our previous studies show that in estrogen-stimulated MCF-7 breast cancer cells, raloxifene at low concentrations (1 or 4 µM) evoked an antiestrogenic effect on collagen biosynthesis and prolidase activity, while an estrogenic effect on gelatinolytic activity. However, at high concentration (10 µM), raloxifene induced estrogenic effects on collagen biosynthesis and prolidase activity, while an antiestrogenic effect on gelatinolytic activity [85]. We also found that, at 10 µM, tamoxifen induced apoptosis in MCF-7 cells. Whether this effect is due to activation of PRODH/POX-dependent ROS formation requires to be explored.

However, recently we have found that, in estrogen-negative MDA-MB-231 breast cancer cells (expressing ERβ), cultured in estradiol-free medium, stimulation of PRODH/POX by troglitazone (TGZ) contributed to apoptosis [98]. The effect was not found in MCF-7 cells, independently of the presence or absence of estradiol in culture medium nor in MDA-MB-231 cells cultured in the medium with estradiol. It has been suggested that the mechanism involves upregulation of PRODH/POX expression (by TGZ) and attenuation of collagen biosynthesis (by eliminating estradiol-induced collagen biosynthesis), that facilitate proline availability for PRODH/POX-dependent apoptosis. The hypothesis was provided that TGZ together with anti-estrogen treatment could be considered as an approach to experimental pharmacotherapy of estrogen-negative breast cancers.

**Figure 6:**
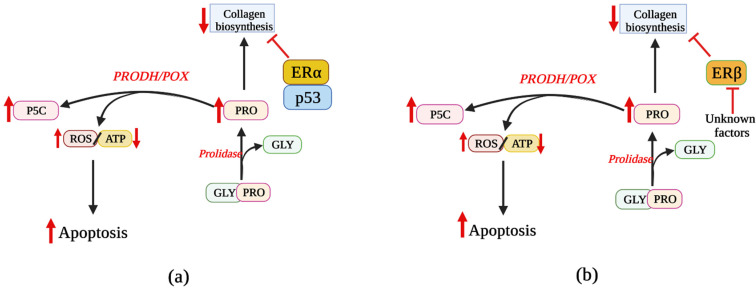
Downregulation of ERα and ERβ facilitates PRODH/POX-dependent apoptosis in breast cancer cells. Activation of both ERs is known to stimulate collagen biosynthesis utilizing proline as a substrate for PRODH/POX-dependent functions. (**a**) since ERα has the ability to form a complex with p53 [66], the process diminishes the potential of ERα to stimulate collagen biosynthesis contributing to an increase in proline concentration, facilitating PRODH/POX-dependent apoptosis. (**b**) The same effect could be achieved by eliminating ERβ or its inhibition by unknown factors [66,90]. P5C—pyrroline-5-carboxylate; PRODH/POX—proline dehydrogenase/proline oxidase; ROS—reactive oxygen species; GLY—glycine; PRO—proline; ATP—Adenosine triphosphate; ER—estrogen receptor.

The above data suggest that ERs are involved in PRODH/POX-dependent apoptosis; however, the complexity of the mechanism of these processes requires further study. Nevertheless, based on the cited facts, it seems that blocking the function of both estrogen receptors promotes PRODH/POX-dependent apoptosis in breast cancer cells. The hypothetical mechanism of this process is shown in Figure 6.

## 7. Conclusions

The hypothesis that estrogens affect PRODH/POX-dependent apoptosis is based on the studies showing estrogen-induced utilization of PRODH/POX substrate (proline) for collagen biosynthesis [99,100]. In this way, estrogens stimulate proline utilization for protein synthesis, limiting its availability as a substrate for PRODH/POX-dependent apoptosis. However, estrogens may differentially affect PRODH/POX-induced functions dependently on the ERβ/ERα status. It seems that ERα has anti-apoptotic potential through antagonizing P53-dependent apoptosis, inducing the expression of HIF-1α, PPARγ, and prolidase. ERβ evokes opposite effects. The data suggest that PRODH/POX-induced apoptosis is dependent on ER status in breast cancer cells.

Therefore, in further studies on antiestrogen therapy, PRODH/POX could be considered as a target enzyme. Since tamoxifen is the only endocrine agent with approval for prevention and treatment of ER-positive breast cancers [101], it would be reasonable to perform more clinical studies on PRODH/POX-dependent apoptosis/survival in tamoxifen-treated ER-negative breast cancers.

## Data Availability

Data sharing is not applicable for this article.
